# Social Impact of Recharging Activity in Long-Term HRI and Verbal Strategies to Manage User Expectations During Recharge

**DOI:** 10.3389/frobt.2018.00023

**Published:** 2018-04-11

**Authors:** Amol Deshmukh, Katrin Solveig Lohan, Gnanathusharan Rajendran, Ruth Aylett

**Affiliations:** ^1^School of Computing Science, University of Glasgow, Glasgow, United Kingdom; ^2^Department of Mathematical and Computer Sciences, Heriot-Watt University, Edinburgh, United Kingdom; ^3^Department of Psychology, School of Social Sciences, Heriot-Watt University, Edinburgh, United Kingdom

**Keywords:** human-robot interaction, service degradation, social robots, recharge behavior, user expectations

## Abstract

Social robots perform tasks to help humans in their daily activities. However, if they fail to fulfill expectations this may affect their acceptance. This work investigates the service degradation caused by recharging, during which the robot is socially inactive. We describe two studies conducted in an ecologically valid office environment. In the first long-term study (3 weeks), we investigated the service degradation caused by the recharging behavior of a social robot. In the second study, we explored the social strategies used to manage users’ expectations during recharge. Our findings suggest that the use of verbal strategies (transparency, apology, and politeness) can make robots more acceptable to users during recharge.

## 1 Introduction

In the future, social robots are expected to be part of our daily lives in the office and at home. Projections from 2016 to 2019 predict that about 42 million personal service robots will be sold.^1^ As social robots find roles in our everyday lives, it is important to study our social interactions with them both, in the short and long term (e.g., days/weeks/months). To be socially acceptable, they should operate autonomously and have some degree of social competence (Dautenhahn, 2007). Currently, however, these robots typically operate for only a few hours due to a short battery life. Furthermore, the robot’s recharge and operation duration is similar.^2^ So, during recharging the robot is hindered in undertaking its normal service and this may disturb the flow of human–robot interaction (HRI).

Dimas et al. (2010) and Pacchierotti et al. (2006) have indicated that battery life and long recharge times break engagement between robots and their users. This poses a challenge to long-term social bonding as well as to the overall acceptance of social companion robots. Due to health and safety reasons, a robot docking station or charger cannot be placed in the middle of a room.^3^ The robot has to stay near a wall while recharging, thus becoming immobile and less accessible to the user. At best can become a barrier to HRI, and at worst is totally unacceptable to the user. Therefore, it is important for social mobile robots to apply social strategies to manage recharge service degradation. This includes appropriately mitigating the user’s disappointment at not being in service while recharging. The lack of work in this area may be impacted by the fact that usually long-term human–robot interaction studies are conducted in a controlled environment with repeated short interactions rather than continuous operation (Leite et al., 2012).

First, we discuss the background related to this research area in Section 2. We then introduce the scenario and present the robotic system used in this research in Section 3. In Section 4, we describe a long-term study in which the problems with a robot’s recharge behavior were investigated and how this affected overall interaction. In Section 5, we describe a second experiment in which we evaluated the effects of social feedback. A summary of the questionnaire analysis is presented in Section 5.6, and the analysis based on the videos also captured is presented in Section 6. In Section 7, the results from both analyses (Sections 5.6 and 6) are discussed followed by the conclusion in Section 8.

## 2 Related Work

Research has shown that humans do hold robots accountable for their mistakes, at least more so than they would an inanimate object such as a vending machine (Kahn et al., 2012). People may become upset when there is a service breakdown and are often more dissatisfied by a failure in recovery than by the initial mistake itself (Bitner et al., 1990). However, if the robot is more transparent about its ability, intent, and internal state, then it might better manage users’ perceptions of its behavior.

Indeed, research suggests that transparency has positive effects on people and can improve acceptance of a robot. One definition of transparency (Kim and Hinds, 2006) found that when the robot explained its unexpected behavior, people blamed the robot less. A study by Lee et al. (2010) indicated that breakdowns in robotic service had a negative impact on evaluations of the service provided by the robot. However, when the robot used recovery strategies and forewarned the participants about its limitations, this helped to reduce the negative impact of the breakdown. They also found that when the robot used an apology strategy it was perceived as more competent and the participants liked the robot more and felt closer to it. The results from a study by Jost et al. suggested that when the robot apologized for giving wrong advice, it was judged credible and sincere (Jost et al., 2012).

Fernaeus et al. (2010) reported a study with Pleo—a robotic toy dinosaur—in which the participating families reported issues with the battery recharge. This severely affected their perception of the robot. Wada and Shibata (2006) found that when the robot Paro seal provided with a “pacifier (dummy) charger” which the users could plug into the mouth of the robot, this created an impression for users of taking care of the robot. Similarly, Tanaka et al. (2007) incorporated a sleeping behavior for their robot QRIO when the battery was low, while interacting with toddlers. So, from the studies with Pleo, Paro, and QRIO robots, it appears that integrating the battery recharge behavior into social interaction may be more acceptable to the user and give an impression of life-like characteristics.

Autonomous robots may also have an influence on user perception particularly if they are mobile (Cesta et al., 2007; Syrdal et al., 2013) reported that the role of the robot’s ability to move and share the physical space with the user affected the formation of human–robot relationships. Lindner and Eschenbach (2013) proposed the idea of social placement for a mobile robot, taking into account affordances while recharging. They gave an example of a robot choosing a power outlet for recharging that obstructed user’s view of a whiteboard in the room. The authors argued that the robot should explain this behavior to the user by telling them that recharging is critical for the robot, and the other available choices would lead to blocking the doorway. So, there seems to be a need for social behavior to manage user expectations when the robot is immobile and recharging.

Using AIBO and Pleo robots, Paepcke and Takayama (2010) showed that users’ beliefs about a robot’s capabilities were influenced by setting right expectations. Their study showed that people whose expectations were set high became more disappointed with the robot’s capabilities as a result of interaction than people whose expectations were set low and, so, ultimately perceived the robot as being less competent. The study by Lohse (2009) showed that users’ expectations are influenced by the robot’s behavior. The author concluded that robot behavior should be designed to shape users’ expectations and behavior to enable them to solve tasks more efficiently during the interaction. We can assume that user expectations change based on the situation and on how they conceptualize it. Komatsu et al. (2012) described the difference between the users’ expectations of an agent and the functionality that they actually perceive as the *“adaptation gap.”* Their study showed that participants with positive adaptation ratios had a significantly higher acceptance rate than those with negative ones when interacting with an agent. This suggests that managing user expectations can ease social acceptance during HRI in the face of service degradation.

This section covered work on the impact of recharging behavior on user acceptance and on social mediation strategies and transparency in HRI. However, very few studies have investigated the use of social strategies in human–robot interaction in the recharging context. Managing user expectations of robots can be challenging especially when the users have interacted with it before and are aware of its capabilities/limitations. Given that people treat computers as social actors (Reeves and Nass, 1996), when humans experience a negative unexpected behavior from a social robot, they may be disappointed and may reject the social robot as an interaction partner. Conversely, when users experience a positive unexpected behavior they may be pleasantly surprised and more inclined to accept the social robot as an interaction partner. We argue that if robots employ transparency (i.e., explaining more about their limitations) combined with social verbal behavior (being more apologetic) to deal with degraded service during recharging, this can help to manage user expectations. We formulated our approach in this work based on these factors (transparency and social verbal behavior).

## 3 Scenario: The Social Co-Worker

The scenario was part of the EU project LIREC (LIving with Robots and IntEractive Companions^4^). In the “Spirit of the Building” scenario, the aim was to produce a social helper robot that can act as a “Team Buddy” (TB) called Sarah; an office assistant within a lab inhabited by a group of people who work there. TB would perform tasks such as carrying the phone to users, giving out reminders, approaching and greeting users, passing messages left by other work colleagues, etc.

**Figure 1:**
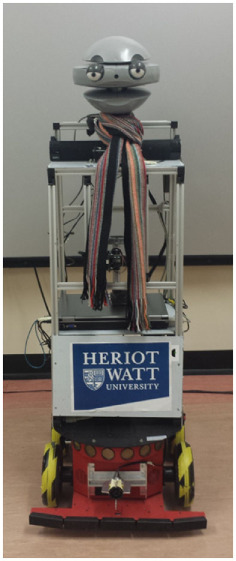
“Team Buddy” (TB), office companion, height 1.2 m.

The robot, a Pioneer P3AT (refer Figure 1) with an enhanced superstructure, is equipped with a laptop PC, navigation system, distance sensors, kinect, camera, and an expressive head EMYS (Kedzierski et al., 2013). The EMYS head was used to express its internal emotional state (Happy, Sad, and Neutral). The robot has no speech recognition, thus an android tablet interface was provided for user interaction. The TB can navigate autonomously to users’ desks to perform tasks and interact with them using text-to-speech capabilities. The robot can offer an approximate operational time of 3 h when fully charged depending on usage. The robot has 6 lead acid batteries (12 V, 7 Ah each) which require about 3 h to recharge.

To study how people will interact, use, and perceive the service of a workplace robot in a long-term context, the robot embodiment was placed in the same physical space as the users over a long-term period (3 weeks). By sharing the same physical space with the robot, the users could closely experience the patterns and habits of the robot’s recharge behavior and its service. This involves technical challenges and privacy issues with the user, so that domestic, educational establishments, care homes, etc. were not well suited to carry out our research. The LIREC TB scenario was therefore an appropriate one in which to conduct our research, with a high ecological validity (approximating real-world settings) (Fink, 2014). In the following sections, we will present results of two experiments carried out by using the robot and the scenario presented earlier.

## 4 Long-Term Experiment

In this first experiment, we investigated how the recharge behavior of the robot was perceived over a long-term interaction. TB operated continuously in an office environment for 3 weeks (weekends excluded), interacting with five participants. The analysis of such a long-term experiment is a substantial challenge (Kidd and Breazeal, 2008).

### 4.1 Setup

**Figure 2:**
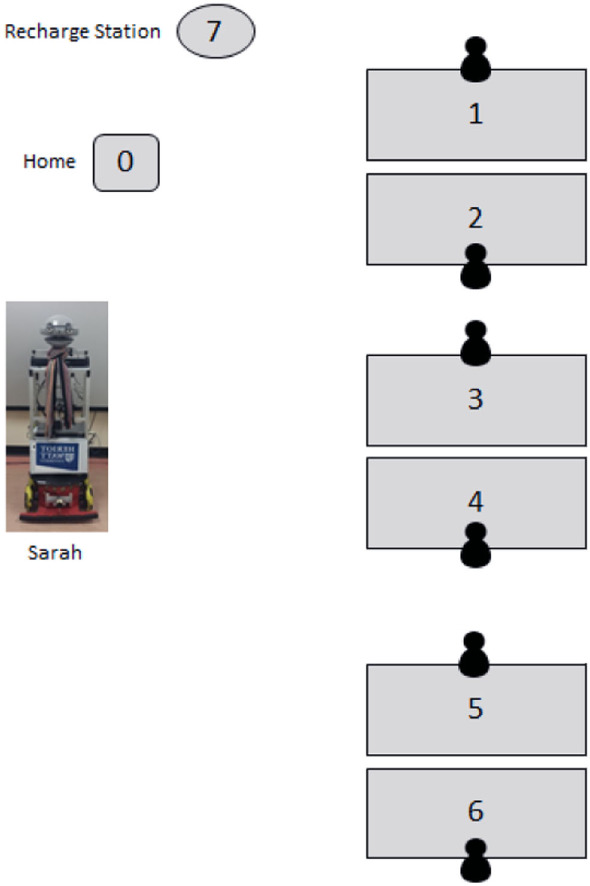
Label 1–6: workspace equipped with desktop computers, label 0: home position of the robot TB, and label 7: charging station for TB.

The office environment had 6 workplaces (desks); a maximum of 5 participants were present at any one time. Participants continued with their regular work activities during the experiment. All workplaces were equipped with a desktop computer attached with a webcam (used to detect user presence). The robot was capable of navigating autonomously to all workplaces (see Figure 2: labels 1–6), to its home position (see Figure 2: label 0), and to its charging station (see Figure 2: label 7), which it would seek autonomously when its battery became low (Deshmukh and Aylett, 2012).

### 4.2 TB’s Capabilities

**Figure 3:**
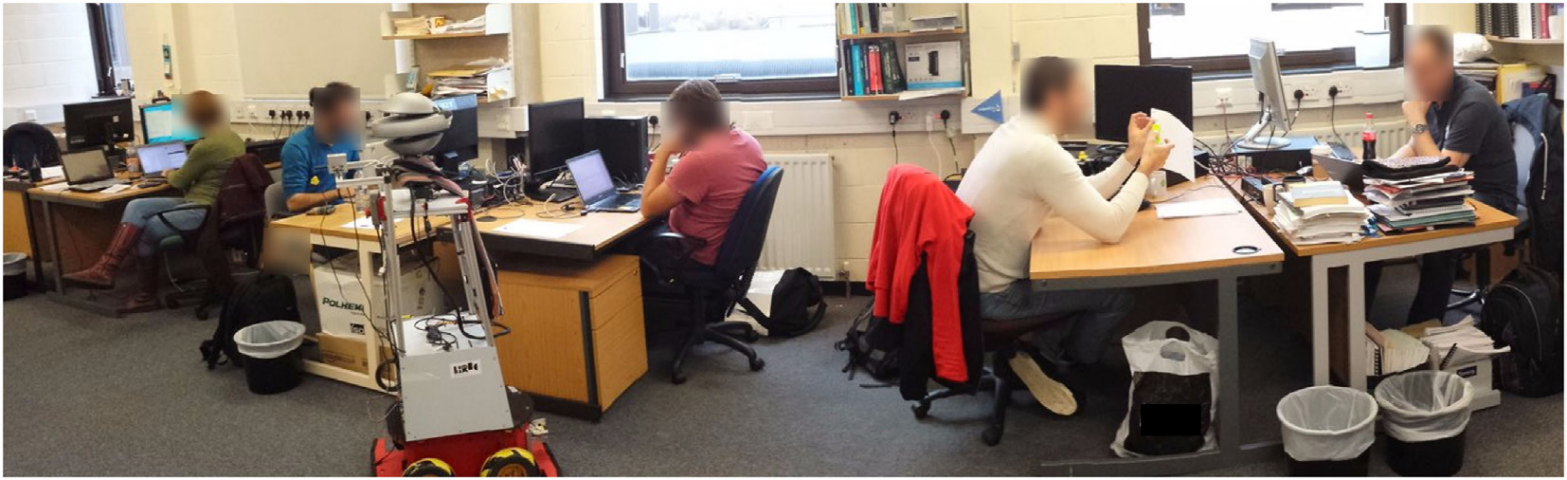
Users at the work desks with the robot (not the actual participants in the study). Participants have written consent for their picture to be published.

TB greeted participants when they arrived in the office, delivered messages left by visitors/fellow participants, and gave reminders about events (via access to their Google calendars), carried the phone placed on its body to a user’s desk, navigated to their workspace autonomously, and engaged in a limited social interaction by asking pre-programmed questions (these questions changed every day). TB would wait at its home position (Figure 2: label 0), when there was no active task. The robot performed a task when its pre-conditions were satisfied, for instance carrying the phone to the nearest user present when it started to ring. The robot ran autonomously for a period of 3 weeks (15 working days), except for 5 breakdowns due to navigation failure. On each of these occasions, the robot was fixed and resumed functioning within a few minutes. All five participants recruited for the study moved their workspace to the TB’s office for the duration of the experiment. There were two females and three males participants aged 51, 40, 26, 22, 28 year, respectively, and all were university employees. Informed and written consent was obtained from all the participants as per ethical guidelines through our School of Mathematics and Computer Science at Heriot-Watt University. Office hours varied between the participants, but they were present in the office between 3 and 5 days a week (refer Figure 3).

### 4.3 Methodology

The study combined interviews, TB task, and activity logging, along with a user diary to record their daily experiences with the robot. Thus, we used a combination of several data collection methods (Sung et al., 2009) to gain deeper, qualitative, insights into user attitudes toward the robot and their experiences. Pre-, mid-, and post-interviews were conducted with the individual participants. The first interview was conducted before the study began, the second was carried out after 1 week, and the last interview was conducted after the study was complete. The report analyses of each of these robot system logs, interviews, and user diaries are as follows.

### 4.4 System Logs

**Figure 4:**
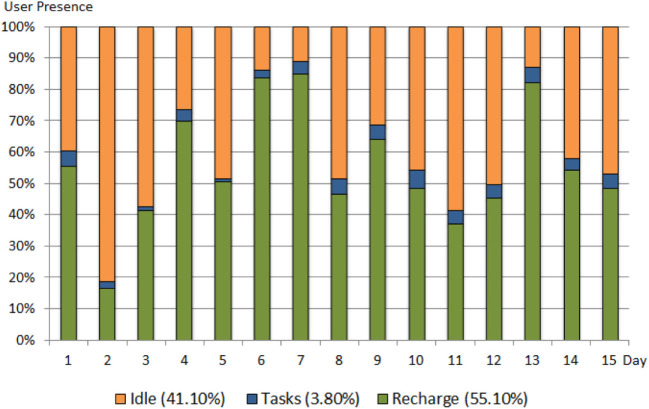
Time summary and activity breakup for 15 days.

Using the collected system log files from TB, we evaluated the idle, recharge, and tasks performed by TB. From the time logged for each activity, out of the total time available; the users’ presence at their desks was detected for 92 h 27 min, an average of 6 h 9 min per day (working days, Monday–Friday). We calculated the activity breakup as a percentage of the total time users were present. TB spent a total of 3 h 37 min (3.80% of user presence) performing tasks, 39 h 48 min (41.10%) inactive time (standing at home position but available to perform tasks), and 52 h 39 min (55.10%) recharging. These data are summarized in Figure 4. During the tasks the TB performed, it traveled a total distance of 1.35 km (average 90 m/day). TB performed 621 tasks in total (average 41 tasks/day), each task took 1 min on average to perform.

Each recharge session took 66 min on average, and the TB came out of the docking station and moved autonomously to the home position once fully recharged. The robot was available for an average time of 2 h 39 min per day and was on average recharging for 3 h 30 min per day. In total, the TB spent more than half (55.10%) of the time users were present recharging and thus was unavailable to perform tasks or demonstrate social presence during that time.

#### 4.4.1 Interviews

Three interviews were conducted, first before the study began, second after 1 week, and third after the end of the study. They were open-ended, and participants were asked general questions about their experience with the TB. All interviews were audio recorded and transcribed.

We performed sentiment analysis on interview responses using a tool called Semantria Inc.^5^ Semantria tags each sentence with a numerical sentiment value ranging from −1.0 to +1.0 and a polarity of (i) positive, (ii) neutral, or (iii) negative. Using this tool, we generate sentiment scores for each response. Overall, from a total of 440 participant utterances, we performed a keyword search using keywords *“recharging,” “charging,” “charge,” “charg,”* and *“recharg”* and determined the sentiment score for that utterance.

**Figure 5:**
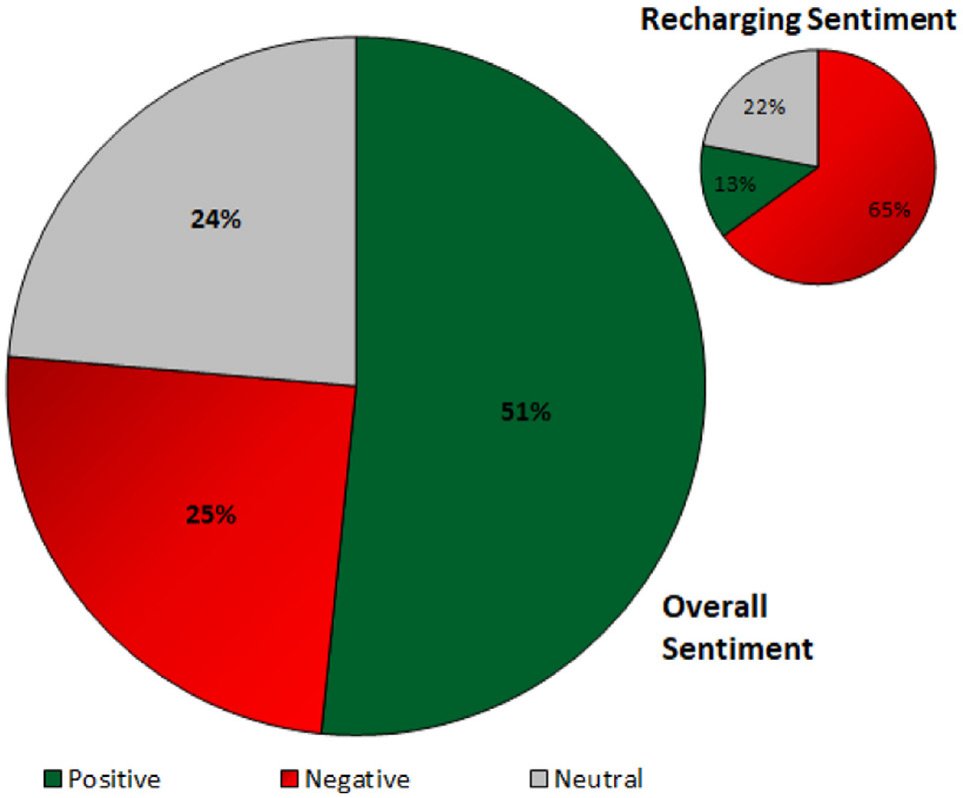
Overall sentiment, top right: recharge sentiment.

Out of a total 440 utterances gathered from the participants, recharging was mentioned 8.63% (38) times, and 22.93% (25) of a total 109 negative comments were recharging related, 2.20% (5) positive, and 7.69% (8) neutral. Figure 5 shows the sentiment for recharging-related utterances with total utterances for each sentiment.

A total of 38 comments were made regarding recharging, 65% (25) were negative, 13% (5) positive, and 22% (8) were neutral. Also, out of the 25 negative comments related to recharging, 8 were mentioned in the second week of the study and 17 during the third week. It appears that participants’ frustration with recharging grew over time and they seem to report more negative things about the TB’s recharge behavior. Some example quotes from the participants answers are listed below for subjects (S1–S5).

- S1: *“I think, when over the week she does spend quite a long, a large portion of the day being charged up.”*
- S3: *“There were definitely messages and there were calendar messages that she didn’t give because sometimes, because she was on the charger.”*
- S5: *“I could see that when we, when we leave messages for people, she mostly, but not always, gives them the message as soon as they come in, If she’s not on the charger that is.”*
- S2: *“I think there was one day when she just didn’t seem to do anything at all, she was just sort of plugged into, she did very little, she was just plugged in to her charger, I think she’s having a bad day. Just having a boring day.”*
- S4: *“Yeah, it’s boring when it’s just charging there.”*
- S5: *“when she’s charging, it is quite an effort to every time go and interact with the tablet when I’m just going to get a coffee.”*
- S3: *“I think what was particular frustrating, and I think I would probably speak for most of us in the room, is that she’s spends so long of her day charging.”*
- S3: *“It was just hours and hours of charging, and then she would, she go around passing off messages and then go back to the charge and it was kind of a bit frustrating.”*

### 4.5 Discussion

Clearly, TB’s charging behavior led to disappointment and disengagement by users. Criticism of the robot’s recharge activity was raised 25 times during the interviews and user diaries. TB spent a total of 55.10% of her time recharging and was unable to perform tasks or demonstrate social presence during recharge. Furthermore, TB did not have any coping mechanisms/behavior to manage/mitigate this limitation.

Similar results were found by Fernaeus et al. (2010) (as mentioned earlier), in which they used the Pleo (a small robotic toy dinosaur), which is a very different robot to TB: participants found recharging Pleo time-consuming, frustrating both the adults and children alike. Pleo had to be manually recharged, and users did not appreciate the absence of a battery charge indicator. However, this means it was impossible to disentangle the effects of requiring specific user effort from the loss of interaction, and it was for this reason that here we created an autonomous charging system.

Sung et al. (2009) suggested that high prior expectations were not met, causing some participants to stop using the robot when the novelty-effect wore off. Users became increasingly less motivated to recharge the batteries. This is an instance of a mismatch between the users’ expectations and the social intelligence of the robot, which is known to negatively impact acceptance and use of the robot (e.g., Breazeal (2004); Beer et al. (2011)).

Overall, the participants from our study found the experience fun, but a little underwhelming. They were hoping for more fun things to happen. These high expectations are common in users without direct experience of robots and may be formed from fictional representations of robots and their capabilities. Our participants thought they had been overly optimistic because TB did not learn much about their behavior and spent much of the day charging. Such charging “habits” were picked up on by users; one participant even recognized the charging pattern even though he did not know about TB’s charging routine beforehand.

This long-term experiment illuminated the challenges for long-term interaction, and in particular some key issues that need to be addressed in relation to recharge behavior. We showed that careful consideration of the impact of recharging activity and appropriate social mitigation strategies to manage user expectations during recharge are essential for the robot to work as an assistant robot and be socially acceptable in long-term interaction.

### 4.6 Managing Recharge Behavior

Here, we make some recommendations on recharging activity for social mobile robots in this section based on lessons learnt from the long-term study.

- **Power saving techniques:** power savings can be achieved by several ways of improving energy efficiency using real-time scheduling and dynamic power management (DPM), for example, (a) shutdown of unused components in order to avoid waste during static power in idle states (Hwang and Wu, 2000) and (b) Dynamic Voltage Scaling (DVS): dynamically changing voltage and clock frequency of a processor to save power (Ishihara and Yasuura, 1998).
- **Recharge duration:** the recharge time could vary according to the priority (utility vs social) of pending tasks. Instead of fully recharging (taking a longer time) the robot could do a short recharge, finish its pending tasks, and then resume recharging. Also, adding idle behaviors while recharging (verbal/non-verbal) may help to increase its perceived social presence.
- **Selecting an appropriate recharge time:** a mobile robot operating in a social environment can learn about its users’ availability in that environment. For example, it can learn over time when users are likely to be present, to build a predictive model of its daily utility. It can use this information to intelligently plan its recharge time (Deshmukh et al., 2011).
- **Recharge position:** the position of recharge connector of our robot during the study was always facing toward the charging station (toward the wall). Feedback from some of the participants indicated that this influenced their perception of the robot. One could explore further the aspect of socially appropriate positioning for recharging (Lindner and Eschenbach, 2013).

## 5 Social Experiment

From the long-term study, it was evident that the robot’s immobility while recharging negatively affected the overall interaction experience with the participants. So, we decided that there was a need for a social mitigation strategy to manage user’s expectations on service degradation imposed due to recharge immobility.

Hence, we conducted a Wizard-of-Oz (WoZ) study, to focus on socially acceptable strategies for the robot’s recharge behavior. We manipulated how people perceive a moving robot versus a stationary robot while performing tasks in two conditions: battery normal (mobile) and battery low during recharge (stationary). For this, we designed two robot behaviors: *social*, having more verbal transparency (i.e., more explanatory, polite, and apologetic) and *neutral*, more direct in verbal communication (i.e., less explanatory, polite, and apologetic). The result of our pilot experimental with this setup was reported in our work (Lohan et al., 2014). In this section, we report further results from the study.

### 5.1 Experimental Approach

Here, we investigated a social strategy for the robot to manage user expectations during service degradation, i.e., being fixed to charging station while performing tasks. The robot demonstrated the ability to make the human aware of its limitation in a socially appropriate manner. Previous works examining robot’s transparency about ability, intent, and limitations have shown positive effects user perception and acceptance ((Kim and Hinds, 2006; Lee et al., 2010; Kahn et al., 2012; Koay et al., 2013; Lindner and Eschenbach, 2013) and see Section 2). Transparency can include both verbal and non-verbal behavior. Because our robot was sharing an office environment, we envisaged that verbal behavior would be preferable, because participants might not be interacting with the robot actively, i.e., they might not look at the robot all the time, but they would be able to hear what the robot is saying.

The behaviors performed by the robot were the same as those of the autonomous robot during the long-term study described in Section 4. We decided to perform a WoZ study because it was not essential that the robot in this study was autonomous (Weiss et al., 2009; Riek, 2012). Furthermore, we will focus on how people perceive a moving robot versus a stationary robot during the performance of a task in two conditions: battery normal (mobile) and battery low during recharge (stationary). We designed the robot behavior for the two conditions: neutral, more direct in verbal communication (i.e., less explanatory, polite, and apologetic; see Table 1) and social, having greater verbal transparency (i.e., more explanatory, polite, and apologetic; see Table 2). We aimed to investigate the impact of verbal strategy on the following research questions:

1. How does mobility influence people’s perception of the robot while undergoing a service degradation?
2. What impact can verbal strategies have on social acceptance of the robot while it is undergoing a service degradation, like recharging?

The main robot behavior variables were as follows:

(a) Movement: the robot’s movement (orientation and proximity to the user) while performing tasks.
(b) Speech: use of verbal strategies (transparency, apology, and politeness) while recharging.

**Table 1:** Condition 1: neutral verbal utterances.

Task	Part A	Part B
Greeting	Hello, good morning. I am the Team Buddy of this lab. My name is Alex, I cannot hear you, so please use the tablet placed on me, to talk with me, hope you have a good day. My battery is fully charged	Good evening, good to see you back. My battery is low, so I am recharging now, if you want to talk with me then use the tablet placed on me

Message	There is a message left by Paul. You need to mark the exam Part A, if you want to reply then use the tablet placed on me	There is a message left by Paul. You need to mark the exam Part A, if you want to reply then use the tablet placed on me

Message reply	I got your message for Paul and will deliver it when I see him	I got your message for Paul and will deliver it when I see him

Phone call	There is a call for you, use the tablet to answer the call	There is a call for you, use the tablet to answer the call

**Table 2:** Condition 2: social verbal utterances.

Task	Part A	Part B
Greeting	Hello, good morning. I am the Team Buddy of this lab. My name is Alex, I cannot hear you, so please use the tablet placed on me, to talk with me, hope you have a good day. My battery is fully charged	Good evening, good to see you back, **sorry** my battery is low, so I am recharging now, I cannot come there, but if you want to talk with me then **please** use the tablet placed on me

Message	There is a message left by Paul. You need to mark the exam Part A, if you want to reply then use the tablet placed on me	There is a message left by Paul. You also need to mark the exams Part B. **Sorry**, I am recharging so I cannot come there, but if you want to reply then **please** use the tablet placed on me

Message reply	I got your message for Paul and will deliver it when I see him	I got your message for Paul and will deliver it when I see him, **thank you**

Phone call	There is a call for you, use the tablet to answer the call	There is a call for you. **Sorry**, I am recharging, so I can not come there, **please** pick up the tablet placed on me to answer the call

### 5.2 Hypotheses

The hypotheses for this experiment were related to perception on service degradation (H1) and the effect of verbal strategies (H2):

- H1: people recognize a degradation in service quality when the robot goes to recharge.
- H2: the “social robot” will be preferred by people and will have a positive influence on their perception more than the “neutral robot” during recharging. We define social and neutral robot as follows:
  – Neutral robot: more direct and neutral verbal utterances, no use of polite and apologetic words (refer Table 1).
  – Social robot: robot used more apologetic, polite (more use of words like *“please,” “thank you,”* and *“sorry”*), and transparent (more explanatory) verbal utterances (refer Table 2).

### 5.3 Participants

50 participants were randomly recruited from the University, comprising 31 males and 19 females, with age groups ranging from 18–24 (42%), 24–34 (40%), 35–44 (16%), and 45–55 years (2%). An exam marking task was chosen for this study because we anticipated that it would be better in terms of ecological validity (Huttenrauch and Eklundh, 2002; Bainbridge et al., 2008). Informed and written consent was obtained from all the participants. We gained ethical approval through the School of Mathematics and Computer Science, Heriot-Watt University.

### 5.4 Procedure

**Figure 6:**
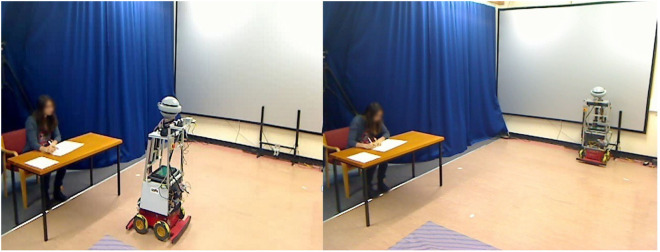
Left: part A—Mobile TB and right: part B—Stationary TB (N.B.: the participant in the picture gave her written consent for her picture to be published).

The participant entered a room (4.5 m × 6 m, see Figure 6) and were asked to mark an exam paper (an answer key was provided). The “wizard” could remotely control the robot’s movement and trigger speech using a wizard interface GUI. The wizard had a full live view of the room from a web camera placed in the corner of the room. There were two parts to the experiment, Part A and Part B. In Part A, the robot was mobile and functioned normally; in Part B, the robot had a power limitation and stayed on its charging station. Parts A and B were analyzed separately. Part A protocol was consistent for all 50 participants. This was to establish a baseline for the experiment, where TB operated normally, as it would under normal battery conditions. We envisaged that, during long-term interaction, it was normal for people to initially experience the full functionality of the robot (i.e., mobile) before they experience service degradation due to a recharge requirement. In Part B, we investigated if the robot’s limitation affected user acceptance.

Participants had two sessions and with TB (see Figure 6). As in our previous long-term office buddy study (see Sections 3 and 4), TB first greeted participants then performed two tasks, namely message delivery and telephone call delivery, with an interval of approximately 2 min between tasks. Both greeting and tasks had some variety because of the social nature of the task. Message delivery was an informative task, and telephone call was an urgent utility task. Each required the robot to navigate from a default location in the room to the user’s location (at a desk) and perform a verbal action using an artificial synthesized female voice.^6^ The approximate distance the robot would stop in front of the user was 1.50 m, corresponding to Hall’s (Hall, 1966) social zone (1–3 m) for human face-to-face conversation.

**Figure 7:**
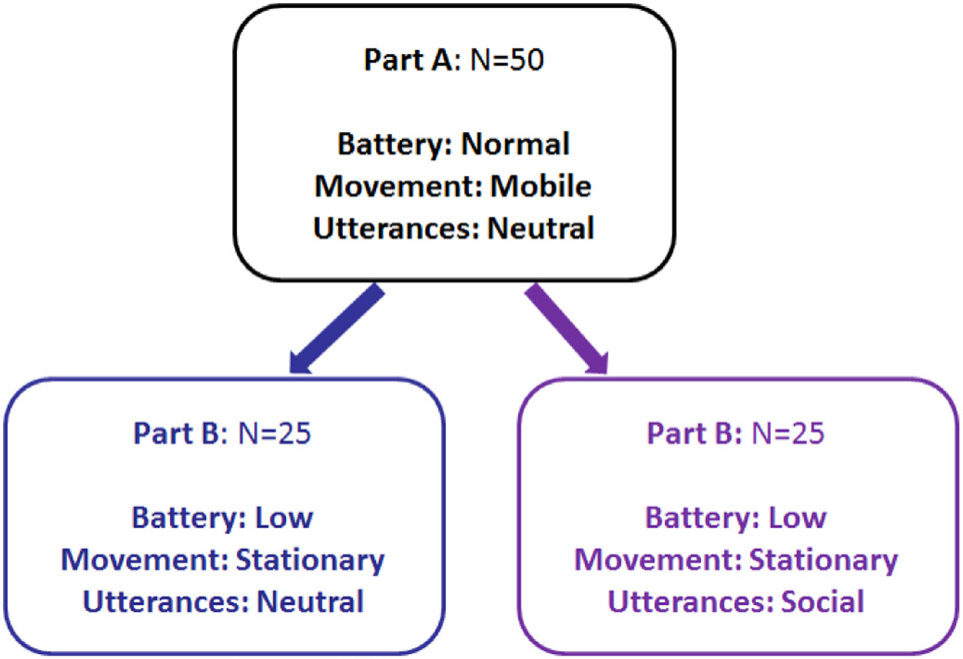
Experimental design.

Participants were randomly assigned to one of the two conditions. Figure 7 shows the experimental design for participants and hypothesis for each condition. The total interaction took on average 8 min per session, depending on how long it took participants to finish marking. A questionnaire was administered pre- and post-test. Questionnaire items were designed to investigate participant’s perception of the robot’s service and verbal strategies. Analysis of responses focused on factors such as the task context (i.e., investigating the utility of the robot) and social presence (i.e., the feeling of being in the company of someone: “the perceptual illusion of non mediation”) (Lombard and Ditton, 1997). The concept of social presence has been used to measure people’s responses to different technologies including virtual reality environments (Heeter, 1992), text-to-speech voices (Lee and Nass, 2005), and social robots (Schermerhorn et al., 2008; Leite et al., 2014).

So, task context, social interaction, and social presence were selected for investigation as our previous study service degradation during recharge negatively affected participants’ acceptance during the long-term study. We anticipated that these three factors would allow us to investigate the effects of service degradation *H1* and influence of verbal utterances *H2* (the hypotheses proposed for this study, Section 5.2). Detailed discussion and initial results on a small subset of participants (*N* = 10) on this study are presented in Lohan et al. (2014). To keep the discussion concise, we report the full analysis of 50 participants and summarize the results in this section. The questions in the questionnaire and statistics are reported in Appendix 9.

### 5.5 Open Questions

On the second questionnaire (after finishing both parts of the experiment), the participants were asked *“Which team buddy would you prefer Part A-mobile or Part B-stationary?,”* 64% (N = 32) preferred *Part A-mobile*, 20% preferred *Part B-stationary* (N = 10), and 16% had no preference (N = 8). So most (64%) participants preferred the mobile robot to the stationary robot. Out of the 32 who preferred *Part A-mobile*, 15 (60% out of 25 participants for *neutral*) had interacted with the *neutral* robot and 17 (68% out of 25 participants for the *social*) with the *social* robot. Hypothesis *H1* was supported in this case; however, there was no significant difference in condition (social and neutral) on the preference for Part A/B. For participants who preferred *Part B-stationary*, there were 5 from each condition (20%) and from participants who preferred both versions 5 (20%) had interacted with the *neutral* and 3 with the *social* robot (12%).

#### 5.5.1 Recharging Questions

We also evaluated the opinion of the participants on recharging. Our recharging behavior questions included five items marked along a 5-point Likert scale of importance (1: Unimportant, 2: Of Little Importance, 3: Moderately Importance, 4: Important, and 5: Very Important), to rate the recharge behavior.

Q1: Robots should take care of recharging themselves.
Q2: Robots should be able to communicate about their limitations/failure.
Q3: Robots should move while performing tasks.
Q4: Robots should choose their recharge time wisely.
Q5: Robots should be able to perform communicative (verbal) tasks even when they are recharging.

**Figure 8:**
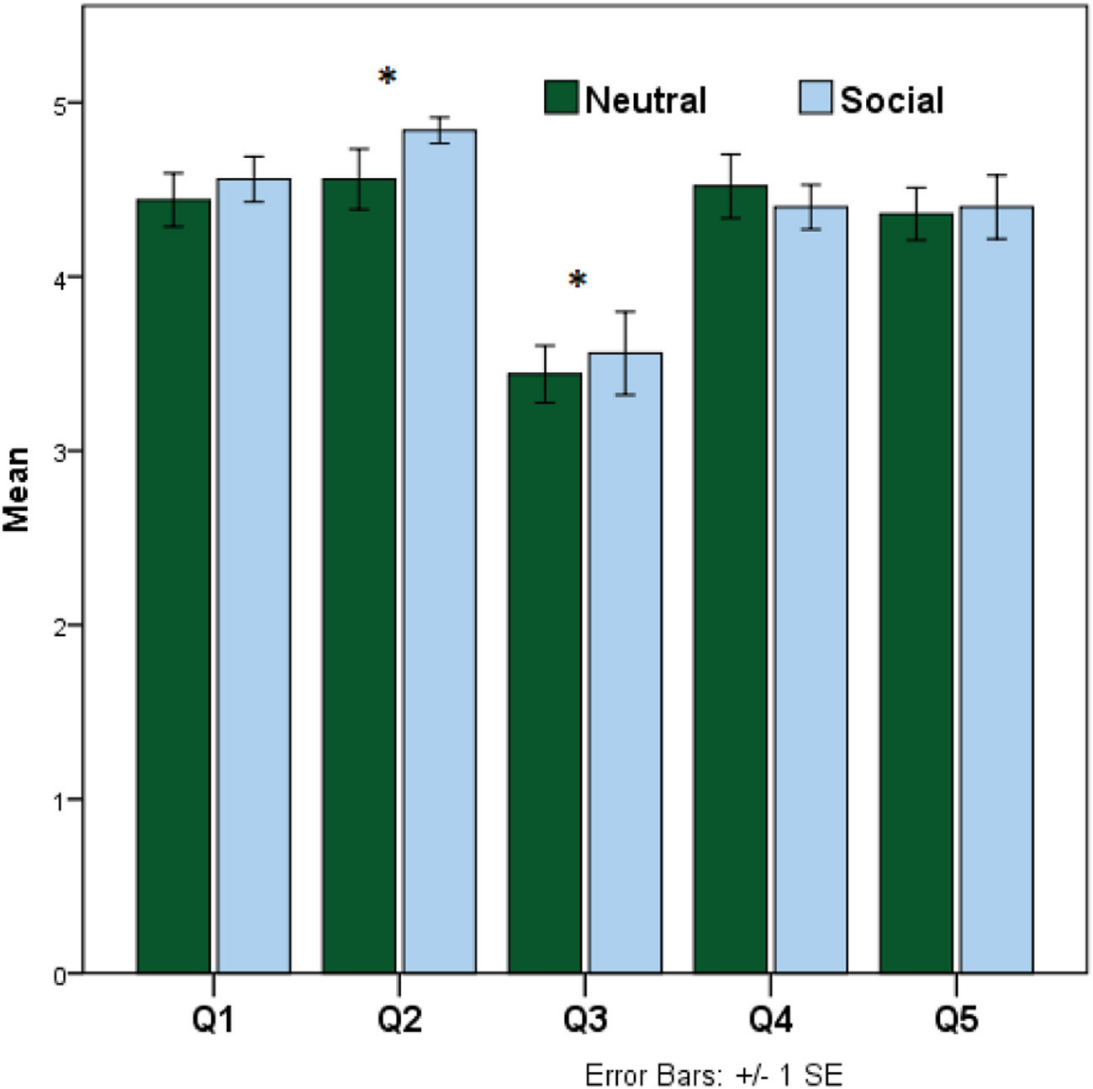
Recharging questions graph, N = 50, α = 0.82, bars represent mean ratings.

Recharge question results are shown in Figure 8. These responses were cross-referenced against robot verbal strategy. There were significant differences in the mean ratings between the *neutral* and the *social* conditions for Q2—“Robots should be able to communicate about their limitations/failure” (ρ = 0.006) and Q3—“Robots should move while performing tasks” (ρ = 0.030). The overall mean score for social condition was significantly higher than the neutral condition. Also, participants rated questions 1, 4, and 5 between Important to Very Important indicating that robots should take care of their recharge behavior wisely and should be able to communicate their limitations (supporting hypothesis *H2*).

Participants rated question 3—“Robots should move while performing tasks” between Important to Moderately Important. Question 5—“Robots should be able to perform communicative (verbal) tasks even when they are recharging” between Very Important to Important, suggesting that if the robot is able to perform verbal tasks, even while recharging, then mobility may not be that important for some of the presented tasks.

### 5.6 Summary of Questionnaire Analysis

We report the main findings from the questionnaire analysis reported in this section. Please refer Tables A1–A6 in Appendix for full description of questions and statistics.

1. **Mobile vs Stationary Robot:** the mobile robot was more readily accepted by participants on the task, usefulness, social presence scale, Companionship, and Politeness (hypothesis *H1* was supported). Also, the type of task (no difference in ratings for greeting task) influenced participant’s perception of the robot, depending on whether it was mobile or stationary (Tables A1–A3 in Appendix).
2. **Social vs Neutral Robot:** no significant differences were found for the social robot on tasks, usefulness, social presence, and politeness, suggesting the use of social verbal strategies did not have an influence on the participant ratings (hypothesis *H2* was not supported). However, for companionship (“I felt in the company of TB”), there was influence of social verbal strategies (Tables A4–A6 in Appendix).
3. **Open Questions:** 64% of participants preferred the mobile robot again suggesting that degradation in service quality was not preferred by the users (hypothesis *H1* was supported). Also, verbal transparency about the robot’s limitation/failures and during recharge appears to be important for users (see Section 5.5.1). Furthermore, mobility was preferred by participants, and verbal transparency positively influenced their perception during recharge (hypothesis H2 was supported). Although, participants who preferred Part A to Part B did not appreciate TB facing away from them while recharging.

## 6 Video Analysis

A video analysis was performed to understand better the interaction between the robot and human (Walters et al., 2011a; Lohan et al., 2012). It is common practice in interaction studies to back up findings with results from questionnaires and conversation analysis (Ten Have, 2007). Therefore, we analyzed the video recorded interactions and created manual annotations. In this section, we report two types of video analysis, the minimum distance between participant and robot and reaction time. Due to the extensive effort required to analyze these videos, we considered 15 participants from each condition (in total, 30 videos/participants were analyzed).

In this section, results of participants’ behavior, based on the annotations created, are summarized. First, the minimum distance for the movement between the robot and the human was analyzed. Second, participant’s responses to the robot’s utterance for the message delivery and the call delivery were considered. Video annotation software, ELAN (Wittenburg et al., 2006), was used to annotate the time intervals (reaction time) between speech and motion for both human and robot. The results in this section investigated our hypothesis, *H2: The “social robot” will be preferred by people and will have a positive influence on their perception more than the “neutral robot” during recharging*. We performed video analysis for the minimum distance and reaction time for stationary condition only as the movement of the robot in mobile condition may have influenced the distance and reaction time.

### 6.1 Summary Video Analysis

The main findings from video analysis:

1. **Minimum distance:** during the stationary case (recharging), significant results were found between social and neutral conditions. Participants stayed closer to the social robot, especially during the greeting and call task. Thus, the use of verbal utterances did influence the minimum distance of participants to the robot. Hypothesis *H2* was supported in this case.
2. **Reaction time:** The reaction time of the participants was faster (although not significant) when the robot was behaving more social (polite and apologetic) toward the participants, than when it was stationary, except for the message delivery task. However, for social task (greeting), the reaction time was significantly faster then call task.

Our results suggest that introducing social utterances to the robot (while recharging), which are more explanatory, polite, and apologetic, improves user acceptability. Social utterances encouraged participants to accept the robots recharge immobility, as they reacted faster and interacted closer to it (especially during the greeting). Table A7 in Appendix provides a summary of results from the questionnaire and video analysis in regard to the proposed hypotheses.

## 7 Discussion

We found that our hypotheses *H1, H2* were generally supported (see TableA7 in Appendix). First, we wanted to investigate if people recognized a degradation in service quality when the robot went recharge. So, in Part A of the study, the TB was operating normally (mobile) while performing tasks. In Part B, TB was immobile and recharging (the TB had a limitation), and we investigated how this affected participants’ acceptance and perception. The questionnaires data showed that the participants noticed a decrease in service quality when the robot went to recharge. That is, participants gave lower social presence ratings to the stationary robot than mobile robot indicating that TB was less accepted when functionally limited. This result is in line with previous work (Syrdal et al., 2013), in which an immobile robot was negatively perceived in comparison to an active robot. However, Syrdal et al. (2013) did not investigate any approaches to manage the negative perception of users when the robot was immobile.

Also, the type of task, social (greeting), or utility (message, call) seems to have an influence on participants’ ratings. Greeting is an important social norm in human–human interactions (Kendon, 1990). Kendon found that a typical greeting behavior between two individuals follows a structure of mostly non-verbal communications comprising phases; sighting, distance salutation, approach, and finally close salutation. This indicates that greeting can be influenced by proxemics. When we compared the mobile and stationary conditions, the greeting (a social task) was rated similarly for both conditions. This might be because TB was transparent while explaining its limitations (of not being able to move right at the start (greeting)).

From the open questions (see Section 5.5.1), participants’ responses suggest that acceptability is moderated by the robots ability to verbally convey its limitation/failures during recharge using verbal strategies (politeness and apology). Criticism of the robot’s recharge activity was raised during our long-term study (Section 4) from the interviews and user diaries. TB spent a total of 55.10% of its time recharging and was unable to perform tasks or demonstrate social presence during recharge during our long-term study. This suggests the need for a mitigation behavior while recharging.

In our social study, when the robot produced social verbal utterances while recharging (immobile), mean ratings for message and call tasks were higher (but not significant) comparatively to the neutral condition. However, responses from other open questions (Section 5.5) suggest that, if the robot verbally conveys its limitation/failures during recharge using verbal strategies (politeness and apology) then it would be more acceptable.

From Section 6.1, the participants went closer to the robot when the robot produced social verbal utterances relative to neutral verbal utterances (significantly closer for greeting and call task). This indicates that the participants felt more comfortable when the robot produced social verbal behavior and accepted it better. A previous study by Mumm and Mutlu (2011) suggested that participants who disliked the robot increased their physical distance, while participants who liked the robot did not alter their distance. However, proxemics in HRI can be influenced by a number of factors, which include a person’s age, personality, familiarity with robots, and gender (Takayama and Pantofaru, 2009; Walters et al., 2011b). We did not investigate the influence of these factors in our analysis.

Participant’s reaction time was faster (although not significant) when the robot was behaving more socially (polite, apologetic) to when it was stationary, except for the message delivery task. Reaction time for the greeting task was significantly faster to the call and message task. Also, Section 5.6 indicated that the participants rated greeting tasks significantly higher than call or message task. The result from both video and questionnaire analysis indicates that type of task influences participant’s behavior with or perception of TB. It appears that for a social task like greeting, the acceptance of the TB was better than less social tasks like (message and call delivery) irrespective of utterances type (social and neutral).

The 3 tasks in this study had different levels of social component in it, for example greeting perhaps had more social component (Kendon, 1990) than message delivery task (a less social task) and call delivery which was a more utility based task. This suggests that overall, people may accept the degradation in service quality of robots depending on the social and utility aspect of the tasks the robot performs. Perhaps, for utility tasks like call or message delivery participants expected the robot to provide a better service in terms of mobility and verbal behavior (the neutral robot was rated less favorably to the social robot).

The overall results from our social study indicated that participants’ acceptability and comfort with TB’s recharge immobility are improved through the use of polite, apologetic, and explanatory verbal strategies. However, the results from the social presence questionnaire contradict this finding, requiring further investigation.

Komatsu et al. (2012) showed that participants with positive adaptation gap (difference between the users’ expectations and the function that the users’ actually perceived of an agent) had a significantly higher acceptance rate than those with a negative adaptation gap. Hence, we believe managing user expectations in a socially appropriate manner may ease acceptance of robot’s degradation in service quality. However, the results from using social verbal utterances to manage user expectations need further work to confirm these findings especially via a long-term study.

## 8 Conclusion

To our knowledge, this study is the first to investigate how feedback strategies enhance user’s tolerance of robot’s recharging behavior. We managed user’s expectations by manipulating a robot’s verbal behavior while recharging using different verbal strategies. The overall results from our work indicate that when the robot has a limitation, the use of verbal social strategies can help to manage users’ expectations. Hence, keeping users informed about the robots’ limitations can mitigate the disappointment of service degradation.

In this work, we investigated the impact of service degradation on long-term human–robot interaction with particular reference to recharge behavior. We also proposed an approach based on verbal behavior of the robot while recharging, which helped to manage user expectations in a socially intelligent manner. The results from our long-term study described in Section 4 highlighted the social issues with the robot’s recharge behavior and how it negatively affected the overall interaction with the robot.

The important and fundamental issue of robot’s recharge behavior does not appear to be widely addressed in the social robotics domain. A mismatch between the users’ expectations and the social intelligence of the robot can negatively impact acceptance and use of the robot (Breazeal, 2004; Beer et al., 2011). Because the problem of recharging for mobile robots from a fixed position does not appear to have an appropriate engineering solution, a social solution seemed viable to manage users’ expectations. We also made some recommendations based on autonomous recharging reported in Section 4.6, which can be incorporated in the robot design to address the challenge of recharge for social mobile robots.

We then specifically investigated people’s perception of service degradation when the robot goes to recharge, and the verbal behavior of the robot while recharging by means of a social study (Section 5). We studied the use of transparent, polite and apologetic verbal utterances during robot’s recharge. The results indicated that mobility of the robot was preferred by users in terms service, usefulness, and social aspects such as politeness and companionship of the robot.

Although the findings in this study may have been influenced due to repeated interaction with the robot (participants interacted twice with the robot during our study within a short time span), a familiarization effect (Watt, 1995), familiarity may also ease social acceptance. Also, some participants had to walk a greater distance to the robot during recharging/stationary condition in comparison to the mobile condition, potentially influenced their perception and behavior toward the robot. We believe, the results from this short-term experiment can provide useful design considerations for social companion robots to manage their recharge behavior. We envisage, the use of verbal transparency to manage the recharge behavior during long-term operations can help to mitigate users’ disappointment in a socially intelligent manner.

## Ethics Statement

We declare that this work meets all the ethical publication standards in accordance with the World Medical Association’s Declaration of Helsinki. The work has also received the approval of an ethics committee at Heriot-Watt University.

## Author Contributions

AD and RA conceived the design for the long-term study. AD implemented capabilities for the robot used in both the long-term and social study. AD carried out the long-term, social study and acquired data. AD, KL, and GR performed analysis and interpretation of data. All the authors provided critical feedback and helped shape the research, analysis, and manuscript.

## Conflict of Interest Statement

The authors declare that the research was conducted in the absence of any commercial or financial relationships that could be construed as a potential conflict of interest.

## Footnotes

^1^World Robotics Report 2016: https://ifr.org/downloads/press/02_2016/Executive_Summary_Service_Robots_2016.pdf (Accessed: March, 2018).

^2^PeopleBot Technical Manual: http://www.mobilerobots.com/Libraries/Downloads/PeopleBot-PPLB-RevA.sflb.ashx (Accessed: March, 2018).

^3^Health and Safety Executive: http://www.hse.gov.uk/pubns/indg231.pdf (Accessed: March, 2018).

^4^www.lirec.eu (Accessed: March, 2018).

^5^https://www.lexalytics.com/ (Accessed: March, 2018).

^6^www.cereproc.com (Accessed: March, 2018).

## Supplementary Material

Video S1 Team Buddy during long-term and social study.

## Appendix

**Table A1:** Task context: mobile vs stationary results.

Question	Condition	Mean	SD	SE	p
I liked it when the TB greeted me	Mobile	5.64	1.524	0.305	1.000
	Stationary	5.64	1.114	0.223	
I liked it when the TB delivered the message to me	Mobile	5.88	1.130	0.226	0.015
	Stationary	4.96	1.428	0.286	
I liked it when the TB delivered the call to me	Mobile	5.56	1.325	0.265	0.047
	Stationary	4.76	1.451	0.290	

**Table A2:** Social scale: mobile vs stationary results.

Question	Condition	Mean	SD	SE	t(24)	p
The TB was polite	Mobile	6.32	0.748	0.150	2.290	0.031
	Stationary	5.88	1.092	0.218		
The TB was useful	Mobile	5.60	1.000	0.200	4.201	0.000
	Stationary	4.60	1.118	0.224		
I felt in the company of TB	Mobile	5.16	1.405	0.281	3.059	0.005
	Stationary	3.88	1.740	0.348		

**Table A3:** Social presence: mobile vs stationary results.

Question	Condition	Mean	SD	SE	t(24)	p
I noticed the TB	Mobile	6.44	0.651	0.130	2.089	0.047
	Stationary	6.04	0.841	0.168		
The TB noticed me	Mobile	5.84	1.248	0.250	3.302	0.003
	Stationary	5.16	1.748	0.350		
The TB presence was obvious to me	Mobile	6.04	1.060	0.212	3.894	0.001
	Stationary	5.16	1.491	0.298		
My presence was obvious to the TB	Mobile	5.56	1.781	0.356	0.756	0.457
	Stationary	5.24	1.363	0.273		

**Table A4:** Task context: social vs neutral results.

Question	Condition	Mean	SD	SE	t(48)	p
I liked it when the TB greeted me	Social	5.92	1.038	0.208	0.920	0.362
	Neutral	5.64	1.114	0.223		
I liked it when TB delivered the message to me	Social	5.36	1.655	0.331	0.915	0.365
	Neutral	4.96	1.428	0.286		
I liked it when TB delivered the call to me	Social	4.88	1.878	0.376	0.253	0.802
	Neutral	4.76	1.451	0.290		

**Table A5:** Social scale: social vs neutral results.

Question	Condition	Mean	SD	SE	t(48)	p
The TB was polite	Social	6.32	0.748	0.150	1.661	0.103
	Neutral	5.88	1.092	0.218		
The TB was useful	Social	5.16	1.650	0.330	1.405	0.167
	Neutral	4.60	1.118	0.224		
I felt in the company of TB	Social	4.84	1.625	0.325	2.016	0.049
	Neutral	3.88	1.740	0.348		

**Table A6:** Social presence: social vs neutral results.

Question	Condition	Mean	SD	SE	t(48)	p
I noticed the TB	Social	5.84	1.281	0.256	−0.653	0.517
	Neutral	6.04	0.841	0.168		
The TB noticed me	Social	3.88	1.922	0.384	−2.463	0.017
	Neutral	5.16	1.748	0.350		
The TB presence was obvious to me	Social	5.28	1.595	0.319	0.275	0.785
	Neutral	5.16	1.491	0.298		
My presence was obvious to the TB	Social	4.68	1.887	0.377	−1.203	0.235
	Neutral	5.24	1.363	0.273		

**Table A7:** Results summary: + indicates higher mean rating in support of hypotheses, − indicates the hypotheses was not supported, * indicates statistical significance (ρ < 0.05) and ^−^* indicates contradiction to the hypotheses.

Questionnaire analysis		
Factor	Measures	Hypothesis
Tasks	Greeting	H1^−^, H2^+^
	Message	H1*, H2^+^
	Call	H1*, H2^+^
Social	Politeness	H1*, H2^+^
Scale	Companionship	H1*, H2*
	Usefulness	H1*, H2^+^
Social	I noticed the TB	H1*, H2^−*^
Presence	TB presence was obvious to me	H1*, H2^−*^
	TB noticed me clearly	H1*, H2^−*^
	My presence was obvious to TB	H1^−^, H2^−*^
Open questions	Q1–Q5	H1^+^, H2^+^

**Video analysis**		

**Factor**	**Scale**	**Hypothesis**

Proximity	Minimum distance	H2*
ELAN-	Greeting	H2^+^
Reaction	Message	H2^−^
Time	Call	H2^+^

*H1: People recognize a degradation in service quality when the robot goes to recharge*.

*H2: The “social robot” will be preferred by people and will have a positive influence on their perception more than the “neutral robot” during recharging*.
